# *In situ* determination of exerted forces in magnetic pulling cytometry

**DOI:** 10.1063/1.5084261

**Published:** 2019-03-14

**Authors:** Joshua Bush, Venkat Maruthamuthu

**Affiliations:** Mechanical & Aerospace Engineering, Old Dominion University, Kaufman 238e, 1 Old Dominion University, Norfolk, Virginia 23529, USA

## Abstract

Localized application of exogenous forces on soft biomaterials and cells is often essential for the study of their response to external mechanical stimuli. Magnetic means of applying forces, particularly those based on permanent magnets and magnetic beads coupled to substrates or cells provide an accessible means of exerting forces of appropriate magnitude. The amount of force exerted, however, is often inferred from calibration performed *ex situ*, with typically similar but different magnetic beads. Here, we construct a simple magnetic tweezer by coupling a pencil-shaped stainless-steel probe to permanent neodymium magnets using a 3D printed adapter. We then demonstrate the *in situ* determination of magnetic bead pulling forces on a super-paramagnetic micro-bead coupled to a soft substrate using traction force microscopy. We determine the force exerted on the magnetic bead by the magnet probe – and thus exerted by the magnetic bead on the soft polyacrylamide substrate – as a function of the distance between the probe tip and the magnetic bead. We also show that we can determine the force exerted on a magnetic bead coupled to a cell by the changes in the traction force exerted by the cell on the soft substrate beneath. We thus demonstrate that forces of nanonewton magnitude can be locally exerted on soft substrates or cells and simultaneously determined using traction force microscopy. Application of this method for the *in situ* measurement of localized exogenous forces exerted on cells can also enable dissection of cellular force transmission pathways.

## INTRODUCTION

Soft biomaterials as well as biological entities such as cells have characteristic mechanical properties that in turn specify how they behave in various mechanical contexts.^1–4^ Localized application of external/exogenous forces can be used to perturb and assess the response of biomaterials or cells at desired locations, as opposed to spatially indiscriminate bulk forces.^5–7^ Several techniques have been utilized for localized force application, including atomic force microscopy,^8,9^ optical tweezers^10^ and magnetic twisting^11,12^ or pulling cytometry.^13–16^ Even though the way these techniques have been applied can be quite varied, the range of forces that can be applied using each technique and the geometrical constraints of each set-up often limits the range of situations to which each technique is suitable.

Magnetic force application in particular can be realized in many configurations:^17,18^ the magnet itself may be an electromagnet or a permanent magnet and the number and strength of magnets used can also vary.^19–21^ In many situations, however, what is desired is just the application of a local force of magnitude high enough to cause an observable response and low enough to not affect sample integrity.^22^ The desired response could be just measurable displacement in the biomaterial or a more complex response such as with cells over longer times. Cells themselves exert (endogenous) forces of the order of a nano-newton (nN) on single adhesions (such as focal adhesions) due to their inherent contractile activity.^23^ Use of permanent magnets to exert forces on biomaterials/cells via magnetic beads provides an accessible means of applying nN scale forces with a limited footprint, as long as a judicious choice of magnet strength, magnetic bead size and type is made.

Pulling forces exerted by permanent or electro-magnets on magnetic beads are often quantified *ex situ* with similar, but different magnetic beads.^18^ Typically, the movement of a magnetic bead (in a viscous fluid like oil) towards the probe tip is imaged and the drag force for creeping flow is computed to quantify the magnetic force as a function of the magnetic probe tip-to-magnetic bead separation. However, actual bead diameters and even shapes can sometimes significantly vary from one magnetic bead to another to various extents within and between lots, depending on the source of the magnetic beads. It is thus desirable to determine magnetic bead pulling forces *in situ*, as they are exerted on the sample. We realize this with traction force microscopy (TFM) first for a polyacrylamide (PAA) gel sample as forces are exerted on a magnetic bead coupled to the gel. We then extend this method for the *in situ* determination of magnetic pulling forces on extra-cellular matrix (ECM) coated magnetic beads coupled to cells adherent on PAA gels.

## MATERIALS AND METHODS

### Cell culture

Madin-Darby Canine Kidney (MDCK II) cells were grown in DMEM (Dulbecco’s modified Eagle’s medium, Corning Inc., Corning, NY) supplemented with L-Glutamine, sodium pyruvate, 1% Penicillin/Streptomycin and 10% Fetal Bovine Serum (FBS) (Corning Inc., Corning, NY), at 37 ^0^C under 5% CO_2_. For plating polyacrylamide (PAA) hydrogels, about 10^3^ cells were plated on a 35 mm culture dish with a hydrogel.

### Preparation of polyacrylamide hydrogel substrates

Polyacrylamide (PAA) gels of shear moduli 100 and 1000 Pa were made with an acrylamide to bis-acrylamide ratio of 3.4%:0.04% and 5.1%:0.12%, respectively. The shear moduli were verified using sphere indentation.^24^ The gels contained red fluorescent beads (diameter 0.8 μm, Spherotech Inc., Lake Forest, IL) as fiducial markers. The PAA gels were polymerized between a silanized and a collagen I or antibody-coated 22 mm x 22 mm glass coverslip (No. 1.5) for an hour. The silanized coverslip was obtained by serial treatment with 2% 3-aminopropyltrimethyoxysilane in isopropanol and 1% glutaraldehyde in water.^25^ The collagen I or antibody coated coverslip was obtained by exposing the coverslip to deep UV light for 5 min, followed by incubation with 0.02 mg/ml collagen I (from rat tail, Corning Inc., Corning, NY) or antibody (donkey anti-Human IgG, Jackson ImmunoResearch, West Grove, PA) followed by washing with PBS (Phosphate Buffered Saline) twice.

### Imaging

A Leica DMi8 epifluorescence microscope (Leica Microsystems, Buffalo Grove, IL) with a Clara cooled CCD camera (Andor Technology, Belfast, Ulster, UK) and an airstream incubator (Nevtek, Williamsville, VA) was used to obtain phase and fluorescence images.

### Magnetic probe fabrication

A magnetically permeable probe of approximate length 7 cm and diameter 0.5 cm was mechanically machined from 416 Stainless Steel (Small Parts, Logansport, IN) with a 1 cm long tapering end with a sharp tip of approximately 500 μm diameter. We reduced the tip diameter to the order of 10 μm via electro-polishing in order to apply substantial localized force. The probe was electropolished in an acidic solution of Phosphoric Acid (Fisher Chemical, Pittsburgh, PA), Sulfuric Acid (Fisher Chemical, Pittsburgh, PA) and deionized water in the ratio of 8:7:5 respectively.^13^ The probe tip was immersed in the acidic solution and 8-20V (higher voltages for faster material removal and lower voltages for finer polishing) was applied for ∼30 second intervals until the desired probe diameter was achieved. The probe was then washed in PBS and stored until needed.

### Magnetic bead coating

Epoxy-coated 4.5 μm magnetic beads (M-450 Dynabeads) were diluted from the stock solution (with 4 x 10^8^ magnetic beads per mL) with PBS at 1:200. The diluted bead samples were incubated separately with Protein A (Prospec, East Brunswick, NJ) or Collagen I for 30 minutes at room temperature and stored at 4 ^0^C.

### Magnetic pulling cytometer setup

A 3-axis micromanipulator (Thorlabs) was used to align and position the magnetic probe tip at precise distances relative to the magnetic bead. An adapter was 3D printed in Acrylonitrile Butadiene Styrene (ABS) to mount two cylindrical (1.25” diameter x 0.0625” thick) neodymium magnets (from K&J Magnetics, Pipersville, PA, with a surface field of 662 Gauss) with their axes perpendicular to that of the probe and affix them to the micromanipulator. The probe tip was positioned directly above the gel surface and the probe tip to bead distance along the x-axis was varied using motor control of the micro-positioner.

### Force application on PAA gel

Protein A coated magnetic beads were incubated with IgG antibody-coated 200 Pa polyacrylamide (PAA) gels for 30 min at room temperature to result in magnetic beads sparsely bound to the PAA gel. The magnetic probe-to-bead separation was varied and corresponding images of the fluorescent micro-beads in the top surface of the PAA gel were recorded.

### Force application on cells

About 1.5 x 10^3^ cells were plated onto a collagen I coated 1 kPa PAA gel and left to adhere overnight in the 37 ^0^C incubator. Thirty minutes prior to the experiment, 10 μL of a suspension of collagen I coated beads (obtained from a 1:200 dilution of the stock solution) were pipetted onto the gel with the cells. The coverslip (with the attached gel) was affixed to a Petri dish using vacuum grease (Dow Corning, Midland, MI) and 10 mM HEPES buffered cell media was added. Once the chamber and the magnetic pulling cytometer were set up on the microscope stage, an isolated cell with a bound magnetic bead was located and the magnetic probe tip was positioned at distances 20 or 10 μm away from the magnetic bead (along a horizontal direction defined as the x-axis) and phase images of the cell and fluorescence images of the micro-beads at the top surface of the PAA gel were acquired for each probe tip-to-magnetic bead separation. Finally, the cell was detached with the addition of 1 mL of 1% SDS to obtain cell-free reference micro-bead images from the top surface of the PAA gel.

### Drag force measurement

A 1:200 magnetic bead dilution was prepared with 1 ml isopropanol (Fisher Chemical, Pittsburgh, PA). 10 μL of this solution was plated on an empty probing chamber and left on the hot plate at 100 °C to evaporate the solvent. A viscous silicone fluid (PDMS base, Sylgard 184, Dow Corning, Midland, MI) of known viscosity (5.1 Pa.s) was then added and left in the vacuum chamber for 30 minutes to degas. The probe end (containing the tip) was then immersed in this viscous medium with suspended magnetic beads. Magnetic beads were located and approached with no more than 3 beads in the frame. The magnetic bead imaging plane was positioned similar to the set-up when forces were applied to PAA gels or cells. Images were captured every ∼0.4 s until the moving magnetic beads reached the probe. Images were then analyzed with CellTracker^26^ (in MATLAB) to determine the instantaneous bead velocity at various distances from the probe. With the instantaneous velocity (v), magnetic bead radius (r) and viscosity of the solution (η), the applied force was calculated using Stokes Law, F=6πrηv.^18^

### Traction force measurements

Cell phase images and microbead fluorescence images (from the top surface of the gel) were first aligned (to correct for drift) using an Image J plugin^27^ and the displacement of the beads were calculated using MATLAB (MathWorks, Natick, MA) with code available at http://www.oceanwave.jp/softwares/mpiv/. Traction forces were then reconstructed from the displacements of the gel surface using Regularized Fourier Transform Traction Cytometry that employs the Boussinesq solution.^28–31^ A binary mask was used to include all traction forces exerted by the magnetic bead on the PAA gel (for magnetic bead on gel experiments) or by the cell on the PAA gel (for magnetic bead on cell on gel experiments) and the vector sum of traction stress times the grid area yielded the net traction force exerted. All binary masks were created using ImageJ and all force calculations mentioned above were using custom-written scripts in MATLAB (MathWorks, Natick, MA).

### Ethics approval

An ethics approval is not required for this study.

## RESULTS

**FIG. 1.**
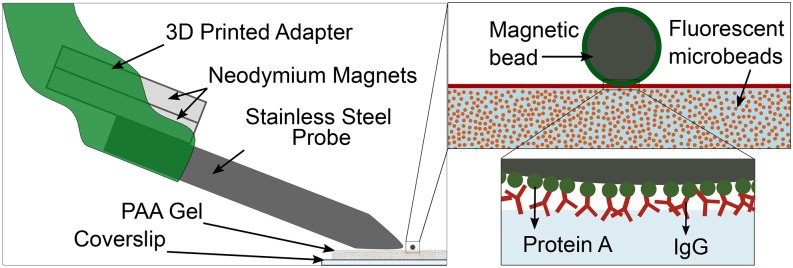
Schematic depiction of the magnetic pulling cytometer set-up (left), showing the 3D printed adapter, permanent neodymium magnets, 416 stainless steel probe, and the ‘magnetic bead on gel’ arrangement with a 4.5 μm superparamagnetic bead bound to a polyacrylamide (PAA) gel attached to a glass coverslip. Insets on the right show the magnetic bead coated in protein A (green) atop the PAA gel coated with IgG (red). Fluorescent beads are embedded within the gel to track the force induced displacements in the gel. Protein A binding to the IgG facilitates magnetic bead binding to the PAA gel in the ‘magnetic bead on gel’ arrangement.

To ultimately apply local forces on cells of the order of nN, which corresponds to the magnitude of cell-generated forces exerted at individual focal adhesions, we constructed a magnetic pulling cytometer (MPC). We coupled permanent magnets to a pencil-shaped stainless-steel probe by 3D printing an adapter that also enabled mounting this set-up on a 3-axis micromanipulator. The pencil-shaped probe was obtained by machining followed by electro-polishing of the tip (see Methods). We first wanted to determine if TFM can be used to determine *in situ* the MPC pulling forces locally applied on PAA gel, a widely used cell culture substrate. To do this, we coupled a protein A coated 4.5 μm diameter superparamagnetic bead onto a PAA gel coated with an IgG antibody (fig. 1). The specific interaction between protein A and the Fc region of the antibody enabled the magnetic bead to bind to the PAA gel (fig. 1).

**FIG. 2.**
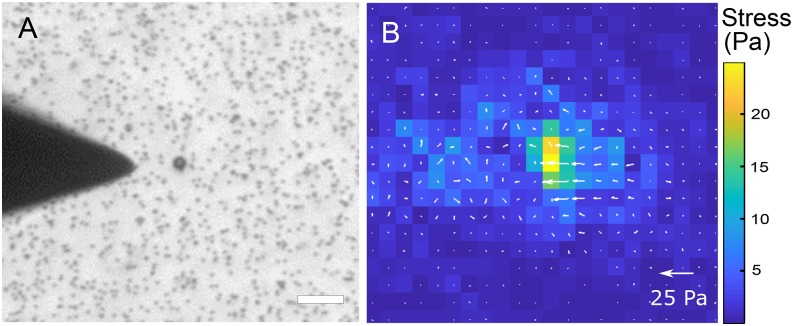
(A) Phase image of the magnetic probe tip near a 4.5 μm magnetic bead bound to a PAA gel. The micro-beads in the PAA gel are also visible. Scale bar is 20 μm. (B) Heat map and vector plot of traction forces exerted on the PAA gel due to the magnetic probe – magnetic bead interaction. Scale bar for the vectors is indicated and that for the heat map is on the right. Note: If the top surface of the substrate is the x-y plane, the indicated stress vectors involve stress components τ_zx_ and τ_zy_.

**FIG. 3.**
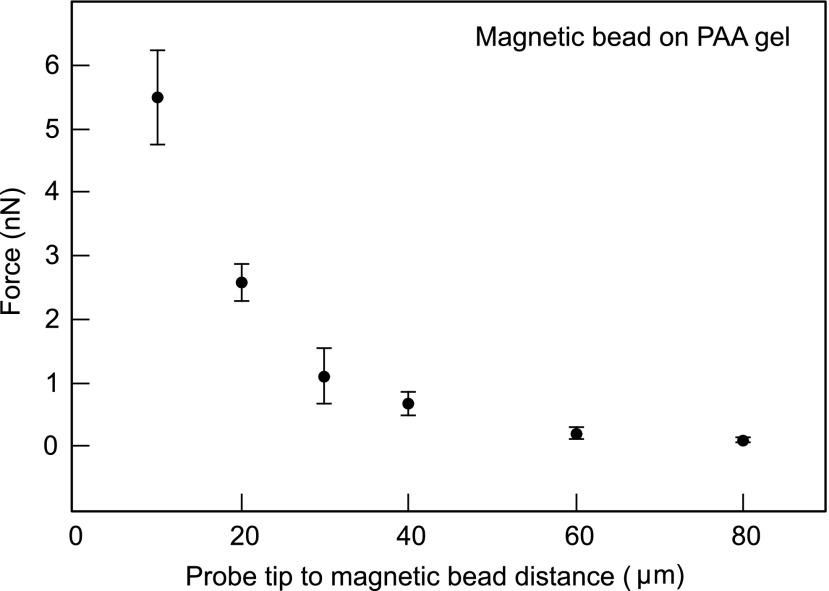
Force (in nN) exerted by the probe tip on the magnetic bead determined by the traction force exerted by the magnetic bead on the PAA gel as a function of the distance between the probe tip and magnetic bead (in μm). Error bars indicate ± standard deviation from two independent experiments.

As the probe tip of the MPC approached the magnetic bead (bound to the PAA gel), the fluorescent micro-beads embedded in the PAA gel were displaced. We used digital image correlation to compute the displacement field and regularized Fourier transform traction cytometry to compute the corresponding traction stress field on the PAA gel (fig. 2). The integrated force exerted by the magnetic bead on the PAA gel (and hence by the MPC on the magnetic bead) was determined as a function of the distance between the probe tip and the (closer) edge of the magnetic bead (fig. 3). For the closest tip-to-bead approach distance of 10 μm, the magnetic pulling force was quantified to be 5.4±0.7 nN. In an alternate set-up, we found that magnetic beads suspended in a viscous fluid (of viscosity 5.1 Pa.s) moved towards the probe tip at a velocity of ∼25 μm/s when ∼15 μm away from the tip. This corresponded to a Stokes drag of ∼5.5 nN, roughly comparable to that obtained from the traction force exerted on the PAA gel. Traction force microscopy thus provides a means of determining the magnetic pulling forces on a specific magnetic bead associated with the sample.

**FIG. 4.**
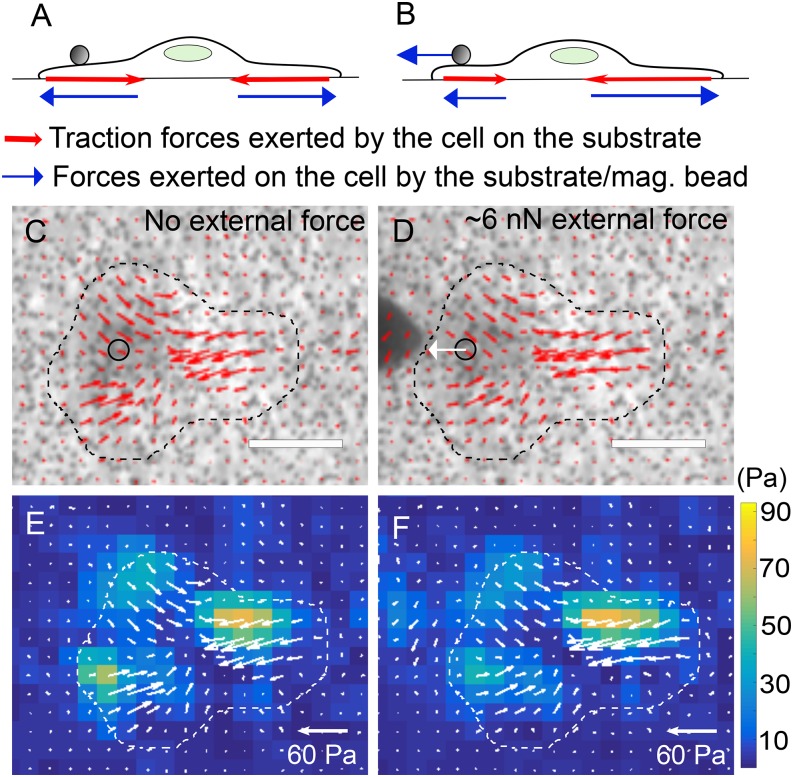
(A, B) Schematic depiction of a cell that is adherent on a substrate, with a magnetic bead attached to it. Traction forces (red) exerted by the cell on the substrate as well as the forces (blue) exerted by the substrate/magnetic bead on the cell are depicted when (A) no external forces are applied on the cell and (B) an external force is applied on the cell via the magnetic bead. (C, D) Phase images of an MDCK cell with a col1-coated 4.5 μm magnetic bead attached to it with (C) no external force and (D) an external force of ∼6 nN applied to it via the magnetic bead. Traction stress vectors (for the traction exerted by the cell on the substrate beneath) are superimposed. Scale bar corresponds to 20 μm. (E, F) Heat map images of the traction stress with superimposed traction stress vectors (white) corresponding to that in (C, D). Heat map scale for traction stress is shown on the right.

We then let an extra-cellular matrix coated magnetic bead adhere to an MDCK cell and wanted to determine if the MPC pulling force exerted on the cell can be determined *in situ* from the traction forces exerted by the cell on the substrate. In the absence of external forces, the net traction force exerted by a cell on the substrate is equal and opposite to the net force exerted by the substrate on the cell and is expected to be essentially zero (within experimental error) (fig. 4A). In the presence of an external force (such as MPC pulling force) also, the net traction force exerted by a cell on the substrate is equal and opposite to the net force exerted by the substrate on the cell. A force balance on the cell shows that the net force exerted by the substrate on the cell has to be equal and opposite to the external force that is exerted on the cell (fig. 4B). Thus, in the presence of an external force, the net traction exerted by the cell yields the external force acting on the cell. Figure 4C and E show the traction stress map underneath the cell in the absence of external forces on the cell. As shown in figure 4D and F, when an external force is applied on the cell, the traction stress map underneath the cell is altered, with traction force magnitude and direction altered in different regions by different extents. At first glance, the traction maps in fig. 4C, E and 4D, F seem similar, but careful inspection shows that both the lengths and orientation of the stress vectors are altered at several locations. The distribution of both the magnitude and orientation of the stress vectors influences the net traction force exerted by the cell and hence leads to significant differences in the net traction force exerted by the cell (in fig. 4C, E vs 4D, F), as quantified below.

**FIG. 5.**
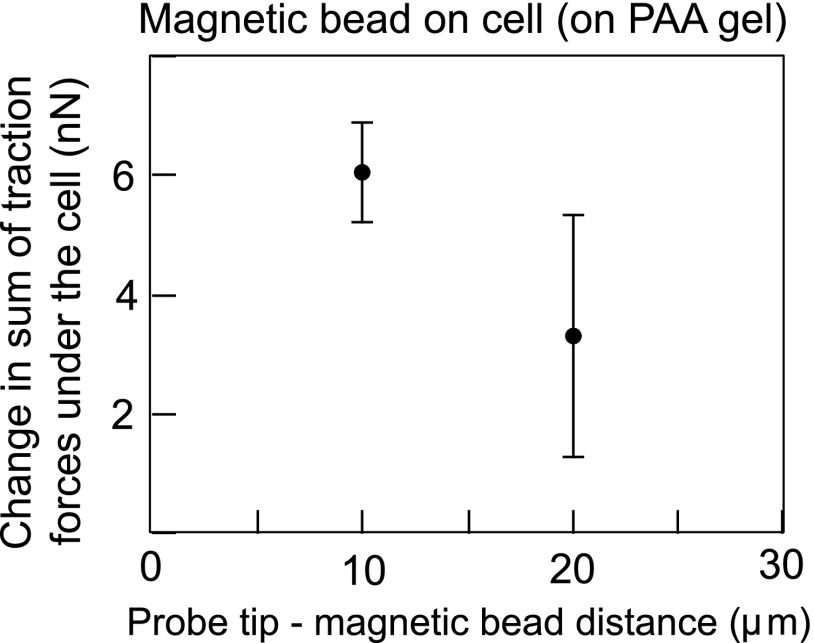
Force (in nN) exerted by the probe tip on the col1-coated magnetic bead bound to a cell determined by the net traction force exerted by the cell on the PAA gel for a distance between the probe tip and magnetic bead of 10 or 20 μm. Error bars indicate ± standard deviation from two independent experiments.

The net traction force exerted by the cell on the substrate is expected to be a read-out of the external force exerted by the MPC probe on the cell (via the magnetic bead), as shown in figure 4B. Thus, we computed the net traction force underneath the cell for probe tip-to-magnetic bead separation distances of 10 or 20 μm – corresponding to the most significant MPC pulling forces in our set-up. As shown in figure 5, we found that the MPC pulling forces computed from the net cell traction forces were 6.0±0.8 nN and 3.3±2.0 nN, corresponding to 10 and 20 μm, respectively. This matched well with the MPC pulling forces obtained directly from the traction forces exerted by the magnetic bead in the ‘magnetic bead on gel’ set-up (fig. 3). Thus, TFM can also be used to determine MPC forces exerted on cells (via magnetic beads) *in situ*.

## DISCUSSION

The use of TFM to determine MPC pulling forces *in situ* offers a major advantage in cases where there are significant variations in magnetic bead properties such as size, shape or magnetization (magnetic dipole moment density). Even when the magnetic bead properties are uniform, significant deviation of the shape from that of a sphere precludes application of the typical Stokes drag equation-based calibration to determine the pulling force. In this case also, TFM offers an alternative means of determining pulling forces as a function of the distance between the probe tip and the magnetic bead. Limitations of the method include the requirement of a substrate with well-defined mechanical properties - more specifically, an isotropic linear elastic material is essential for MPC force measurement using TFM as presented here. Here, we have used PAA gels as they are well characterized isotropic linear elastic materials.^32^ Use of soft silicone gels is also a viable alternative as they are also well-defined soft substrates whose stiffness can be tuned from the sub-kPa to the tens of kPa range.^33^ Another limitation is the need to approach the magnetic bead with the probe tip to a distance of 10s or 100s of micrometers, depending on the strength of the magnet and the dipole moment of the bead. This presents difficulties when the magnetic bead is embedded inside a sample and hence cannot be approached close enough. Also, while large forces (several nN) exerted on the cells (via the magnetic bead) can be deduced using TFM (see Fig. 5), smaller forces (<nN) cannot be reliably deduced due to the effect of noise in TFM.^34^ We do not expect TFM-based force determination to supplant the drag force method of calibration. Rather, we propose that TFM-based force determination is a complementary method that is especially useful in some of the situations outlined above.

We showed here that TFM can be used to obtain MPC pulling forces even for forces exerted on a bead bound to a cell, by measuring the traction forces exerted by the cell on the substrate. However, the stiffness of the substrate over which the cells are cultured needs to be chosen such that (a) the stiffness is high enough that the cells adhere, spread well and exert significant traction forces^35^ and (b) the stiffness is low enough that the cells can deform the gel and the traction forces can be determined without significant noise.^31^ For epithelial cells, this is expected to be in the range of a few kPa and may be higher for cells such as fibroblasts. A potential use of the *in situ* determination of MPC pulling forces on cells from traction forces determined underneath the cells is the mapping of locations underneath the cell to which the external forces are transmitted. Use of beads with higher magnetization and higher resolution imaging can enable the finer determination of regions where the traction forces are significantly altered. Combination with imaging of focal adhesions can help assess the extent to which such force transmission can alter the adhesions at locations distant from the location at which the external force is initially applied.^36^ Such approaches may be essential to understand how mechanical signals mediate cell-level morphological changes and mechanotransduction.
